# Pulmonary Disease Caused by *Mycobacterium marseillense,* Italy

**DOI:** 10.3201/eid2010.140309

**Published:** 2014-10

**Authors:** Antonella Grottola, Pietro Roversi, Anna Fabio, Federico Antenora, Mariagrazia Apice, Sara Tagliazucchi, William Gennari, Giulia Fregni Serpini, Fabio Rumpianesi, Leonardo M. Fabbri, Rita Magnani, Monica Pecorari

**Affiliations:** University Hospital-Policlinico and University of Modena and Reggio Emilia, Modena, Italy

**Keywords:** *Mycobacterium marseillense*, pulmonary disease, immunocompetent, tuberculosis and other mycobacteria, Italy

**To the Editor**: *Mycobacterium marseillense* was recently described as a new species belonging to the *Mycobacterium avium* complex (MAC) (*1*). We describe a case of pulmonary disease caused by *M. marseillense* in an immunocompetent patient. All strains isolated from the patient were preliminarily identified as *M. intracellulare*; however, a retrospective molecular analysis corrected the identification to *M. marseillense*.

In December 2005, a 65-year-old man was admitted to the University Hospital, Modena, Italy, with a 2-week history of fever, cough, and hemoptysis. Physical examination detected diffuse rales, and chest radiographs showed a diffuse nodular opacity and bronchial thickening, confirmed by high-resolution computed tomography (CT) of the chest (Figure, panel A). The patient had experienced several previous episodes of hemoptysis and persistent productive cough since 1998, and tubular bronchiectasis had been detected on previous high-resolution CT images. The patient had a history of thalassemia minor, was HIV negative, and was formerly a mild smoker (10 cigarettes/day for 4 years during his youth). He had no chronic disorders and no history of immunosuppressive-drug or alcohol use.

**Figure:**
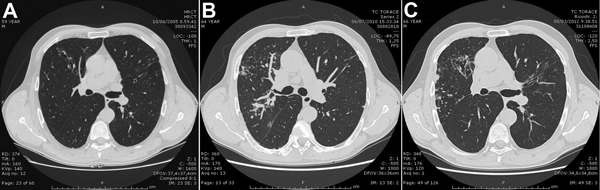
High-resolution computed tomographic chest images of a man with prolonged pulmonary disease caused by *Mycobacterium marseillense,* Italy. A) October 6, 2005. Bilateral bronchiectasis, mainly in the middle lobe and lingula, associated with multiple nodules and middle lobe consolidation. B) June 7, 2010. Increased micronodular opacities, mainly in the right middle and lower lobes, and a worsening of the bronchiectasis. C) August 3, 2013. Persistence of nodular component, cavitation, and wider bronchiectasis with bronchial wall thickening.

Bacterial and fungal cultures and a smear for acid-fast bacilli performed on a bronchoalveolar lavage (BAL) sample were all negative. A nontuberculous mycobacterium strain was isolated by culture and preliminarily identified as *M. intracellulare* by using the GenoType Mycobacterium CM/AS Kit (Hain Lifesciences, Nehren, Germany). At that time, a drug susceptibility test for isoniazid, rifampin, streptomycin, and ethambutol was improperly performed (i.e., was not applicable for MAC) by using the agar proportion method; sensitivity information for macrolides was unavailable. The strain was resistant to ethambutol and susceptible to the other drugs. The physician prescribed rifampin, isoniazid, and amikacin. After remission of fever and hemoptysis and improvement of chronic cough, the patient was discharged from the hospital.

In March 2006, he was readmitted to the hospital for worsening of his condition and onset of side effects associated with rifampin and isoniazid use. The treatment was discontinued and replaced by levofloxacin, terizidone, and azithromycin, which resulted in remission of symptoms. This therapy was continued after hospital discharge.

In 2007, the patient was twice admitted for follow-up and microbiological testing to determine bacteriologic status. All 3 separate sputum samples were negative for mycobacteria, other bacteria, and fungi. However, BAL sample culture results were positive for the same mycobacterium despite continued therapy with levofloxacin, terizidone, and azithromycin.

During 2008, as an investigation of the possibility of persistent excretion of organisms, additional samples were collected 5 times. The sputum cultures were intermittently positive, while the BAL sample cultures were persistently positive.

In May 2009, after the patient had been persistently stable and had negative culture results for 14 months, the antimicrobial drug therapy was stopped. In December 2010, the patient’s only symptom was persistent productive cough; however, the sputum culture was again positive, and high-resolution CT revealed a worsening condition of his lungs (Figure, panel B). A new antimycobacterial drug regimen of ethambutol, rifampin, and azithromycin was started, in accordance with the international guidelines of the American Thoracic Society and the Infectious Diseases Society of America (*2*).

After the patient had received 6 months of therapy, the sputum culture result was again negative. The acid-fast bacilli smear and culture results remained negative until November 2012. The latest treatment resulted in recovery from symptoms and a more stable condition. However, cough and sputum production, although attenuated, persisted despite treatment and negative microbiological test results. High-resolution CT images (Figure, panel C) indicated overall progression of pulmonary involvement from the time of first admission.

In February 2011, on the basis of the known cross-reactivity of the *M. intracellulare* probe with most MAC species when the GenoType Mycobacterium CM/AS assay is used (*3*), we determined the sequences of a portion of the *rpo*B gene and internal transcribed spacer–1 region in 2 strains isolated from sputum in March 2006 and June 2010. All sequences overlapped with *M. marseillense* type strain sequences in GenBank, showing an identity of 100% in internal transcribed spacer–1 with EU266631 and 99.8% (1 mismatch) in *rpo*B with EF584434. 

We therefore show the association of *M. marseillense* infection with pulmonary disease in an immunocompetent patient, helping define the clinical features and natural history of pulmonary disease caused by *M. marseillense*. We cannot assert whether the clinical course is associated with the intrinsic characteristics of *M. marseillense* infection or with the therapeutic regimen, possibly influenced by numerous adverse effects that may have compromised its effectiveness. More careful management in accordance with the American Thoracic Society and the Infectious Diseases Society of America guidelines for management of nontuberculous mycobacterial diseases could have achieved a more effective course of treatment. More case reports of pulmonary disease caused by *M. marseillense* are needed to support our observations and to provide more insight into its clinical picture.

## References

[1] Ben Salah I, Cayrou C, Raoult D, Drancourt M. *Mycobacterium marseillense* sp. nov., *Mycobacterium timonense* sp. nov. and *Mycobacterium bouchedurhonense* sp. nov., members of the *Mycobacterium avium* complex. Int J Syst Evol Microbiol. 2009;59:2803–8. 10.1099/ijs.0.010637-0 19628609

[2] Griffith DE, Aksamit T, Brown-Elliott BA, Catanzaro A, Daley C, Gordin F, An official ATS/IDSA statement: diagnosis, treatment, and prevention of nontuberculous mycobacterial diseases. Am J Respir Crit Care Med. 2007;175:367–416. 10.1164/rccm.200604-571ST 17277290

[3] Tortoli E, Pecorari M, Fabio G, Messinò M, Fabio A. Commercial DNA probes for mycobacteria incorrectly identify a number of less frequently encountered species. J Clin Microbiol. 2010;48:307–10. 10.1128/JCM.01536-09 19906898 PMC2812265

